# The identification of the Romanovs: Can we (finally) put the controversies to rest?

**DOI:** 10.1186/2041-2223-2-20

**Published:** 2011-09-26

**Authors:** Michael D Coble

**Affiliations:** 1 National Institute of Standards and Technology, Applied Genetics Group, 100 Bureau Drive, MS 8314, Gaithersburg, MD 20899-8314, USA; 2 Former Chief of Research, The Armed Forces DNA Identification Laboratory, 1413 Research Boulevard, Rockville, MD 20850, USA

## Abstract

For much of the 20^th ^century the fate of the last Imperial family of Russia, the Romanovs, was a mystery after their execution in 1918. In the mid 1970s the mass grave of the Romanov family (minus two of the children) was discovered and officially exhumed after the fall of the Soviet Union. Forensic DNA testing of the remains in the early 1990s was used to identify the family. Despite the overwhelming evidence for establishing the identity of the Romanov family, a small but vocal number of scientists have tried to raise doubt about the DNA testing during the late 1990s and early 2000s. With the discovery of the two missing Romanov children in 2007, there was an opportunity to re-analyze all of the evidence associated with the case which confirmed the initial DNA testing and brought finality to the mystery. This article will discuss the controversies associated with the Romanov identification and reflect upon the importance of the case to the field of forensic DNA typing over the last 20 years.

## 

In the summer of 2007, three amateur Russian archeologists discovered 44 bone fragments and teeth near the Old Koptyaki Road in Ekaterinburg, Russia (Figures 1 and 2). The discovery was approximately 70 m from the site where the remains of Tsar Nicholas II were discovered about 30 years earlier (Figure 3). I was the Chief of the Research Section at the Armed Forces DNA Identification Laboratory (AFDIL) at the time and was on vacation when the reports from Russia hit the news. I thought it would be unlikely that AFDIL would be involved in the testing, given the large number of ancient DNA laboratories capable of testing the remains now compared to the few laboratories available in the early 1990s, when the first set of remains was recovered.

**Figure 1:**
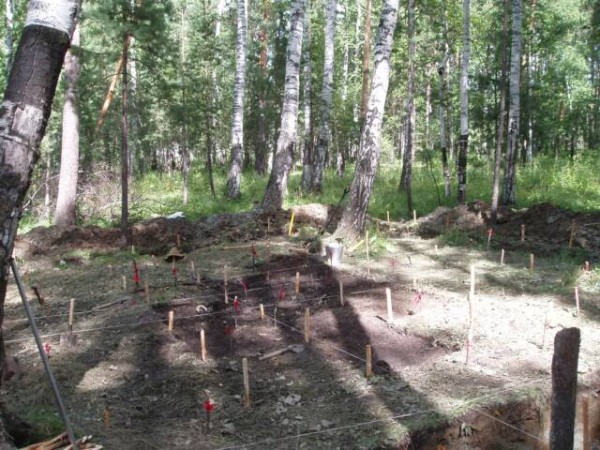
**Excavation of the site where three amateur archeologists discovered the remains of the two missing Romanov children in the summer of 2007**. (Photograph courtesy of Dr Sergei Nikitin and Peter Sarandinaki (http://www.searchfoundationinc.org/).)

**Figure 2:**
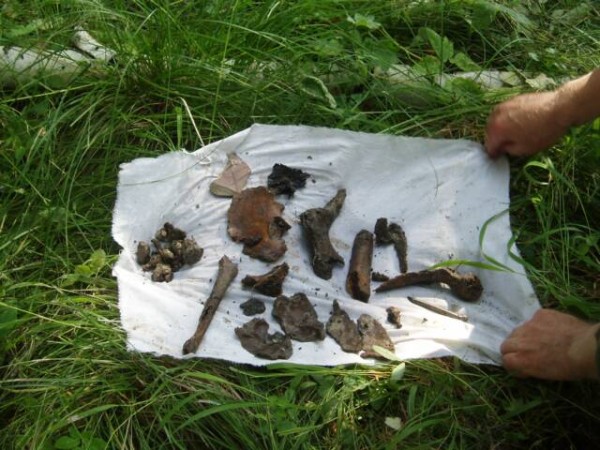
**Skeletal remains and archeological artifacts recovered from the 2007 site**. (Photograph courtesy of Dr Sergei Nikitin and Peter Sarandinaki (http://www.searchfoundationinc.org/).)

**Figure 3:**
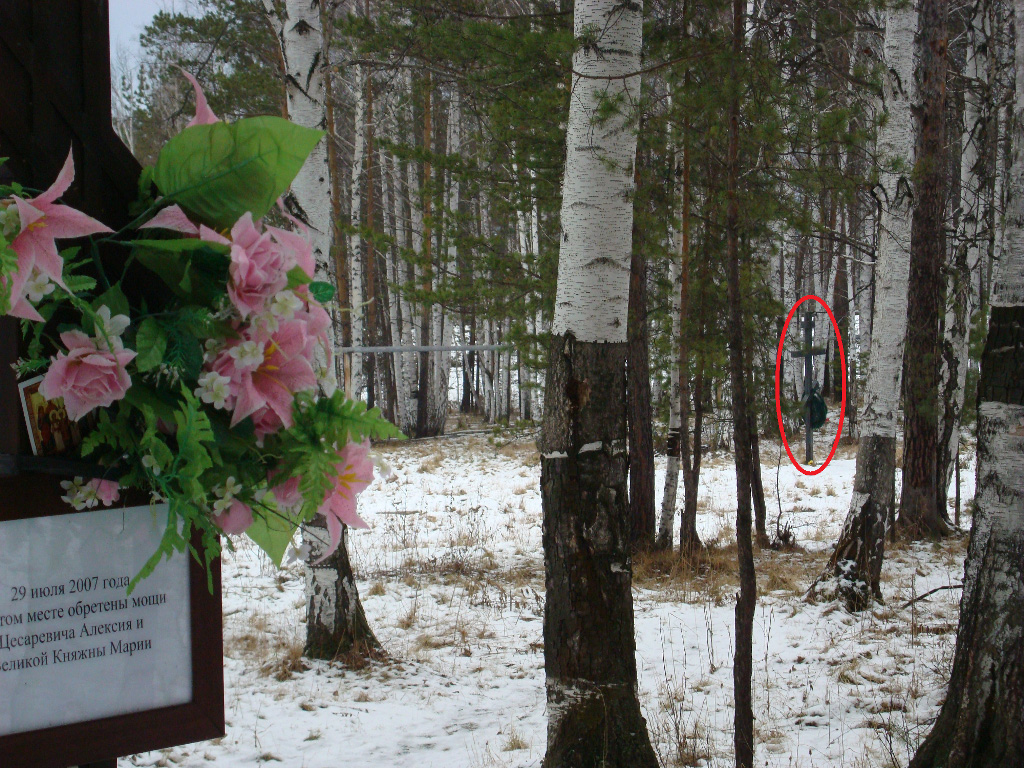
**In 2008, a Russian Orthodox cross was placed at the site of the second grave**. This photograph was taken next to the cross (adorned with flowers, left) at the second grave. A Russian Orthodox cross at the present Romanov Memorial marking the first grave discovered by Dr Avdonin is circled in red and is approximately 70 m away. (Photograph by Michael D Coble.)

About one month or so later, LTC (Dr) Lou Finelli (the laboratory's director) called me in the office to discuss the possibility that AFDIL would be invited in the testing of the remains. Peter Sarandinaki of the Scientific Expedition to Account for the Romanov Children (S.E.A.R.C.H.) Foundation (http://www.searchfoundationinc.org/) had spent years of his life to find the missing Romanov children, and he was able to convince the Russian authorities that AFDIL should be involved in the testing [1].

The eventual fall of Tsar Nicholas II (Figure 4) and the growth of Soviet Communism changed the course of history. I propose that the identification of the Romanov remains was also a defining moment for forensic DNA testing, almost as critical as the first application of "DNA fingerprinting" using restriction fragment length polymorphism technology [2,3] to identify Colin Pitchfork's DNA [4]. The identification of the Romanovs was an important breakthrough in the development and acceptance of forensic autosomal short tandem repeat (STR) and mitochondrial DNA (mtDNA) testing for highly compromised skeletal remains.

**Figure 4:**
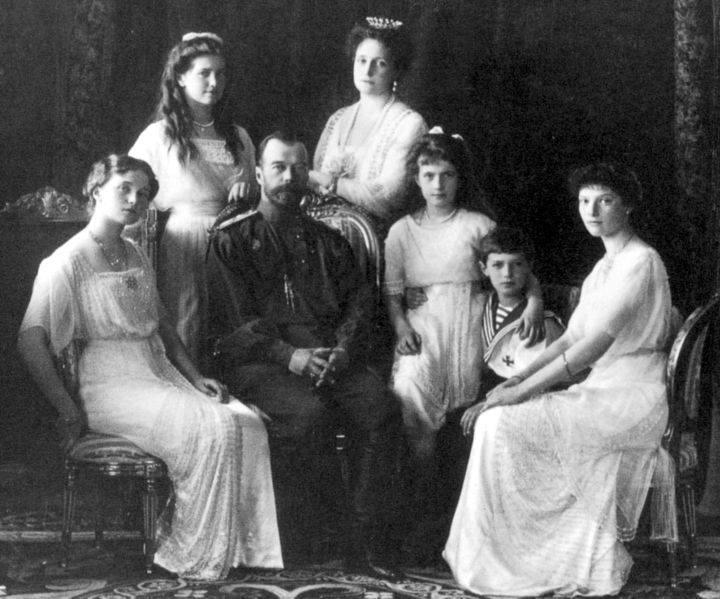
**The last Russian imperial family**. Left to right: Olga, Maria, Nicholas II, Alexandra Fyodorovna, Anastasia, Alexei and Tatiana. Portrait by the Levitsky Studio, Livadiya, Ukraine.

Despite the overwhelming evidence for establishing the identity of the Romanov family, a small but vocal number of scientists have tried to raise doubt about the DNA testing. This doubt perhaps played a role in the Russian Orthodox Church's refusal to accept the identification of the remains in 1998 [5]. With the discovery of the two missing Romanov children in 2007 and the subsequent retesting of the original material, we can now reflect on the forensic DNA controversies surrounding the identification of the Romanovs and gain an appreciation of how much the field of forensic DNA typing has evolved during the past 20 years.

## DNA testing of the first grave

**Figure 5:**
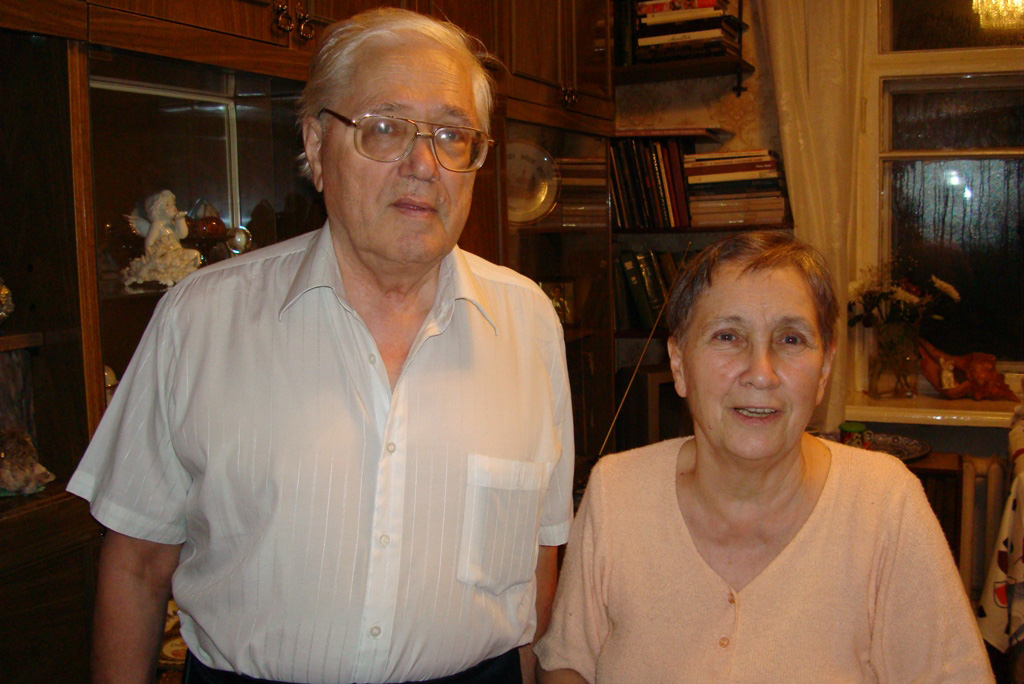
**November 2007 photograph of Dr Alexander Avdonin (left) and his wife Galena**. Dr Avdonin discovered the first mass grave in the 1970s. (Photograph by Michael Coble with permission from the Avdonins.)

In the mid-1970s, Dr Alexander Avdonin (Figure 5) discovered the mass grave containing the imperial family and their loyal staff, with the exception of two of the children: Alexei and Maria. With this discovery, the secret of the disappearance of the Romanovs was no longer a mystery [6]. However, it was not until the fall of the Soviet Union in 1991 that Avdonin and his friend, filmmaker Geli Ryabov, came forward to reveal their secret to the rest of the world. DNA testing of the remains was commissioned by Russian authorities, to be conducted by Russian geneticist Dr Pavel Ivanov and one of the world's preeminent forensic scientists, Dr Peter Gill, at the Forensic Science Service (FSS) in the United Kingdom [6]. Dr Erica Hagelberg from the University of Cambridge was also invited to replicate the findings of Gill and Ivanov [7].

The approach to the DNA testing by Gill *et al. *[7] was twofold, using autosomal STRs and mtDNA sequencing of the two hypervariable regions (HVI and HVII). STR testing was used as a sorting tool to distinguish each skeleton and show the putative familial relationships among the remains. At the time of the DNA testing, autosomal STR markers were in their infancy. The FSS utilized a set of five well-characterized autosomal STRs: vWA, TH01, F13A1, FES/FPS and ACTBP2 (SE33). The first four of these markers were used to create the "quadruplex," one of the first multiplex STR assay kits [8]. Each marker was amplified in singleplex assays for the nine femora recovered in Ekaterinburg (Tsar Nicholas, Tsarina Alexandra Fyodorovna, three of their daughters and four of their servants).

The successful amplification of the remains with the singleplex loci, rather than a megaplex of 16 markers utilized today, was an early preview to the utility of mini-STRs. With the exception of SE33, the loci analyzed by Gill *et al. *[7] had an allele range of approximately 130 to 240 base pairs, making the markers useful for typing degraded DNA. All markers were successfully genotyped, with the exception of SE33 for two of the remains (the servants). It would be several years later before the community would "rediscover" the utility of smaller polymerase chain reaction (PCR) amplicons for challenged samples. It is also worth noting that Gill *et al. *[7] used increased sensitivity techniques (for example, increased PCR amplification cycles) to generate the STR genotypes of each individual. This was necessary to generate results from these challenged samples. The STR profiles were used to sort, sex and show familial relationships among the Romanov family members.

Having established the family unit with STR testing, mtDNA testing was used next to link the tsar and tsarina to living maternal relatives. Forensic mtDNA testing, like autosomal STRs, was also in its infancy in the early 1990s. The comparison of the tsarina's haplotype to her distant cousin, HRH Prince Philip, was an exact match in HVI and HVII.

For the tsar, the mtDNA testing was not as easy and straightforward. In the femur of the tsar, Gill *et al. *[7] discovered the presence of a C/T point heteroplasmy at position 16,169. In the mid-1980s, mtDNA heteroplasmy was believed to be extremely rare or nonexistent [9] and was therefore a "controversial" observation, necessitating further investigations in which cloning was used to confirm the presence of two different DNA strands. Data from family studies of patients with Leber's hereditary optic neuropathy conducted in the late 1980s and early 1990s [10,11] suggested mtDNA heteroplasmy to be more common. Bendall *et al. *[12] found that, among twins, point heteroplasmy was indeed widespread and proposed that the lack of detection in previous studies was likely due to the limitation of detecting low-level variants using sequencing chemistry of the day.

The maternal references for Nicholas, Princess Xenia Cheremeteff Sfiri and the Duke of Fife were homoplasmic at position 16,169 for the T nucleotide base. Realizing that this "apparently rare" condition of heteroplasmy in the tsar would create consternation among scientists and nonscientists alike, Pavel Ivanov had a discussion with Dr Victor Weedn, then director of AFDIL, during the International Symposium on Human Identification (the "Promega" meeting) to conduct additional testing at AFDIL the following year (P Ivanov and V Weedn, personal communication).

The Russian authorities exhumed the remains of Nicholas's brother, Georgii, for additional mtDNA testing. Ivanov *et al. *[13] gave clear and convincing evidence that the 16,169 heteroplasmy found in the tsar was in fact authentic, since Georgii had the same point heteroplasmy, although at a ratio different from that of the tsar. We now have the luxury of a growing body of knowledge about mtDNA heteroplasmy. A PubMed search of "human mtDNA heteroplasmy" returned over 630 articles published over the past 23 years. We now know that heteroplasmy is not a "rare condition" but easily identified if the detection method is sensitive enough [14]. The Romanov heteroplasmy has become the textbook example of mtDNA heteroplasmy [15-17].

In addition to the skeletal elements tested by Gill *et al. *[7], an antemortem piece of evidence from Nicholas II was tested. As a young man in 1891, Nicholas was sent on a global tour by his father, Tsar Alexander III. While in Otsu, Japan, Nicholas was attacked with a saber by a would-be assassin. Nicholas suffered two blows to the right side of his head, above the ear, before the assassin was subdued. A handkerchief used to stop the bleeding was preserved and eventually held in a Japanese museum. Ivanov attempted to develop a clean DNA profile from a piece of the material during his 1995 visit to AFDIL. Ivanov hoped to develop a profile from the material to match with the results from the skeletal remains. However, he had no success obtaining a profile from this material (P Ivanov, personal communication).

## The controversies ensue

With the completion of the scientific investigations, the identification of the Romanovs was concluded. Although two sets of skeletal remains belonging to Alexei and one of his sisters were missing from the mass grave, the forensic results were very conclusive in identifying the Romanov family and their servants. Unfortunately, this was not the end of the story. Before the ink was barely dry on the two reports identifying the last Russian imperial family, the "doubters" were ready to deliver their response. A very thorough review of the efforts to discredit the forensic DNA testing, including an understanding of the political furor during this time period (which I will avoid here) can be found in an article by Nelipa and Azar [18,19] (also available at http://www.oocities.org/mushkah/Nagai.html).

## The Nagai investigations

The first challenge to the Romanov results came from a Japanese scientist, Tatsuo Nagai, in 1997. Using the handkerchief museum artifact from the failed assassination of Nicholas in Japan, Nagai declared that he was able to develop a profile from the cloth and that this profile did not match the results of Gill *et al. *[7]. This work was never peer-reviewed or published [18,19]; however, it gave ammunition to the "doubters" that the remains found in Ekaterinburg were in fact counterfeits. In 2008, two different Russian laboratories performed DNA testing on the shirt that Nicholas wore during that attack [1,20]. A full profile of Nicholas's autosomal DNA and Y-STR haplotype matched the postmortem profile developed from the skeletal remains.

In 1999, a collaborative effort of Nagai and a Russian scientist, Dr Vyacheslav Popov, again tried to disprove the Romanov testing by examining hair samples that were purported to be from Georgii Romanov. Nagai and colleagues obtained and sequenced 25 hair samples from Georgii and compared the data to the mtDNA sequence established by Gill *et al. *[7]. The Japanese researchers determined that, on the basis of the sequence data generated, there was no match to the Nicholas mtDNA sequence, especially at position 16,169, where they found only the revised Cambridge Reference Sequence (rCRS [21]) variant with no hint of heteroplasmy. This paper was published [22], albeit in a Japanese-language journal, *Igaku to Seibutsugaku*. Although I have no knowledge of or ability to translate Japanese, I was kindly given a portion of the publication translated into English by Mr Junichi Hayashi at the request of Margarita Nelipa and Helen Azar for their report [18,19]. I also obtained a copy of the paper from the US National Library of Medicine (Bethesda, MD, USA).

The relevant sequence information from the Nagai *et al. *[22] data requires no understanding of Japanese to decipher and is summarized in Table 1. It is clear that the hair sample they sequenced was contaminated. Seven nucleotide positions have a mixture of two bases: 16,093 T/C, 16,278 C/T, 16,298 T/C, 16,325 T/C, 16,327 C/T, 16,356 T/C and 16,362 T/C. The only reported "difference" listed in the HVII region is 320C, which matches the rCRS. Nagai *et al. *[22] erroneously reported that Ivanov *et al. *[13] had observed a transition at nucleotide position 320 (C-T). The translation of the conclusions from the study is summarized as follows: (1) We observed no heteroplasmy at 16,169, (2) we found seven different heteroplasmic positions in the hair from Georgii Romanov, and (3) therefore, doubt exists regarding the authenticity of the remains recovered from Ekaterinburg.

**Table 1:** Summary of sequence data^a^

		Nagai *et al.*	G. Romanov	
np	rCRS	**sequence**^**b**^	**haplotype**^**c**^	Hg C1a
16,093	T	**C/T**	T	T
16,126	T	T	C	T
16,169	C	C	C/T	C
				
16,223	C	**T**	C	**T**
				
16,278	C	**C/T**	C	C
16,294	C	C	T	C
16,296	C	C	T	C
				
16,298	T	**C/T**	T	**C**
				
16325	T	**C/T**	T	**C**
				
16,327	C	**C/T**	C	**T**
				
16,356	T	**C/T**	T	**C**
				
16,362	T	**C/T**	T	T
73	A	n.r.	G	A
93	A	n.r.	A	G
249	A	n.r.	A	del
263	A	n.r.	G	G
290 + 291	AA	n.r.	AA	del
315.1	-	n.r.	C	C
320	C	C	C*	C

^a^Sequence data are from Nagai *et al. *[22], who erroneously reported 320T for the G Romanov haplotype from Ivanov *et al*.'s paper [13]. np = nucleotide position, n.r. = not reported. ^b^As reported in Table 1 by Nagai *et al. *[22]. ^c^As reported by Ivanov *et al. *[13].

A closer examination of the sequencing results generated by the Nagai investigation is interesting. By using the HaploGrep algorithm [23], an online program that determines mtDNA haplogroups based on the mtDNA phylogeny from PhyloTree [24], one finds that five of the eight SNPs (16,223 C-T, 16,298 T/C, 16,325 T/C, 16,327 C/T and 16,356 T/C) are associated with the Asian haplogroup (Hg) C1a (Table 1). The remaining three "heteroplasmic" SNPs (16,093 T/C, 16,278 C/T and 16,362 T/C) are potential private mutations associated with the C1a individual or DNA templates sequenced from other contaminants. It is especially confounding that no differences in HVII were reported in the Nagai *et al*. study [22]. This includes the lack of the ubiquitous 263A-G and 315.1C differences from the rCRS. Given the apparent contamination of an HgC1a individual in the hair samples, one would also expect to observe differences at nucleotide positions 93, 249 (deletion) and 290/291 (double-deletion) in HVII (Table 1).

Most experienced forensic researchers would categorize the results of Nagai *et al. *[22] as inconclusive because of contamination, and they would draw no conclusions from the results. Melton *et al. *[25] synthesized data from nearly five years of casework hair samples and found that aged hairs showed a decrease in their ability to generate a full profile and an increased chance of researchers' observing mixtures. Nagai *et al. *[22] offered no explanation for the high number of apparent "heteroplasmies" in the hair of Georgii Romanov [18,19,22]. Their only conclusion was that the remains recovered in Ekaterinburg were not those of Tsar Nicholas II. It is unfortunate that individuals (to this day) offer this as evidence to refute the work of Gill *et al. *[7] and Ivanov *et al. *[13].

On 17 July 1998, 80 years to the day after the Romanov family's execution, the skeletal remains recovered in Ekaterinburg were interred at the Peter and Paul Fortress in St Petersburg. On the basis of the uncertainty of the DNA results, the Russian Orthodox Church has not recognized the remains as those of the Romanov family.

## The Knight investigation

The second challenge to the scientific evidence of the Romanov remains came from the laboratory of Dr Alec Knight at Stanford University. The Knight *et al*. study was peer-reviewed and published in the journal *Annals of Human Biology *[26]. The crux of the criticism in the Knight study was the initial Romanov testing and the nested PCR method that the Gill team used to amplify the mtDNA. Gill *et al*. [7 used a nested PCR method in which the entire control region was first amplified with 30 PCR cycles. An aliquot of this PCR product was then amplified in a second round of amplification that targeted shorter amplicons within the control region.

The Knight group argued that the amplification of such a relatively large fragment (approximately 1,200 bp) from a degraded sample, such as the samples from the Ekaterinburg mass grave, was not possible and that the results obtained by Gill *et al. *were most likely contamination from modern DNA. The Knight group obtained a finger bone purported to be from Empress Alexandra Fyodorovna's sister Elisabeth (also known as "Ella"). Ella was married to Nicholas's Uncle Sergei, who was assassinated in 1905. Following the death of her husband, Ella devoted the rest of her life to the Russian Orthodox Church by becoming a nun in 1909 to help the poor of Moscow. She, along with several other Romanov relatives, was executed by the Bolsheviks the day after the execution of Nicholas's family in 1918 [27]. Her body was recovered by villagers near Alapaevsk and was sent to a Russian Orthodox Church in Jerusalem.

Using the bone sample from Ella, the researchers argued that they were unable to amplify three large DNA fragments (437, 466 and 1,179 bp) but were able to successfully amplify two short DNA fragments (108 and 128 bp) from this sample. This "lack of replication" for the larger DNA fragments, the Knight team argued, was evidence that the Gill team had not amplified the endogenous DNA of the Ekaterinburg remains. Since the modern-day reference sample used by Gill *et al. *[7] (HRH Prince Philip) did not match their sequence from Ella, Knight *et al. *[26] concluded that the initial DNA testing of the Romanov remains was wrong.

Like Nagai and Popov, Knight *et al. *[26] found a mixture in the mtDNA sequence data. Rather than declare an inconclusive result based on the data, the Knight group conducted additional cloning experiments to separate the mixed mtDNA fragments. The study determined that the "consensus" mtDNA haplotypes from their molecular cloning experiments were the authentic sequence of Ella [26]. The "consensus of the clones" approach would not be an appropriate method by which to determine an authentic sequence, especially in a forensic investigation. If a forensic sample is contaminated, it is impossible to know *a priori *if the "major" component is endogenous DNA or exogenous contamination. A rebuttal of the conclusions of the Knight investigation was published in the journal *Science *[28,29]. Knight *et al. *[30] responded with many of the same arguments previously enumerated [31].

## Concluding thoughts

Both Gill *et al. *[7] and Ivanov *et al. *[13] evaluated the evidence in their investigations using a Bayesian approach to evaluate the weight of the evidence under two opposing hypotheses (likelihood ratio): (1) These are the remains of the Romanovs, and (2) these are not the remains of the Romanovs.

The statistical analysis of the data was anchored on a verified control sample which was not controversial: HRH Prince Philip is a distant maternal cousin of the Empress Alexandra Fyodorovna. Nagai and Knight did not evaluate their results within this framework. Instead, each investigator evaluated the proposition that the hair was from Georgii and the bone was from Ella, without consideration of an alternative hypothesis that the hair and bone were from an unknown individual. In fact, Knight *et al. *[26] speculated that if their sample truly matched the sequence of HRH Prince Philip and was simply not detected among their clones because of degradation, then Gill's results were likely contaminated by a person who (amazingly) just so happened to be in the same maternal lineage as HRH Prince Philip!

During our work on the identification of Alexei and his sister (exactly which sister could be a debate worth considering for another day), we had two scientists from the forensic laboratory in Ekaterinburg to observe and assist us in the testing of the remains. Our diligent translator, Alexy Zacarin, was there with us every day to bridge the language divide. Alexy, a very spiritual man, once told me that he believed the ground had to hold the remains of the Romanovs until the time came for the Russian people to accept what had happened and for the science to identify the bodies. Perhaps in hindsight it was fortunate that the remains of the two missing children were found 30 years after the mass grave was discovered by Alexander Avdonin in 1977. Technical advances in forensic DNA typing have finally brought closure to the identification of the entire Romanov family.

It is has taken nearly 20 years to test, retest, replicate and confirm the Romanov remains with mtDNA, autosomal STRs and Y-STRs. Our field has grown and matured since the original DNA testing of the Romanov remains. The Gill *et al. *investigation [7] was the first forensic case to show the utility of mtDNA testing of old degraded material. It laid the foundations of the quality assurance methodology: ultraclean rooms, typing analysts and replication of results. It was also the first example of low-template DNA testing with STRs using enhanced cycle numbers. The initial work from 1993 to 1996 [7,13] was a watershed moment for DNA testing, and despite the feeble attempts to discredit these studies with contaminated data, the results have withstood the test of time throughout scientific advances.

It is now time to put this controversy to rest.

## Abbreviations

SNP: single-nucleotide polymorphism; mtDNA: mitochondrial DNA.

## Competing interests

The author declares that they have no competing interests.
